# Ulceration and Perforation of the Heart

**Published:** 1831

**Authors:** 


### LXVI

### Ulceration and Perforation of the Heart.

[Hôtel Dieu.]

Case. Margaret F.——, aged 51 years, was received into the Hôtel Dieu on the 8th March, 1831, complaining of obscure symptoms of indigestion, and being unable to give any satisfactory account of her feelings or ailments. Her tongue was pale and slightly furred—pulse regular, and a little accelerated—bowels torpid, and without tenderness of abdomen on pressure. Leeches to the epigastrium—ptisans—soup for aliment. The patient remained in nearly the same state during the ten days which she passed in the hospital, and the want of prominency or intensity in the symptoms caused her case to be little attended to. On the 19th of the same month, however, she was found dead in her bed, having made no noise or struggle to awake those who slept next to her.

*Dissection.* The pericardium was found adherent to the heart in several places, and in the interspaces was much coagulated blood. The pericardium and the blood being removed, a deep ulceration was seen in the posterior and middle part of the left ventricle, completely perforating the parietes. The internal orifice was ascertained to be ulcerated and ragged like the exterior orifice. The muscular substance of the heart did not appear to be unnaturally soft, except for a small space around the perforation. There was no other appreciable lesion in the organ—*Lancette Francaise.*

---

